# Interactions between Genetic Variants in *AMH* and *AMHR2* May Modify Age at Natural Menopause

**DOI:** 10.1371/journal.pone.0059819

**Published:** 2013-03-27

**Authors:** Marieke G. M. Braem, Marlies Voorhuis, Yvonne T. van der Schouw, Petra H. M. Peeters, Leo J. Schouten, Marinus J. C. Eijkemans, Frank J. Broekmans, N. Charlotte Onland-Moret

**Affiliations:** 1 Julius Center for Health Sciences and Primary Care, University Medical Center Utrecht, Utrecht, The Netherlands; 2 Department of Reproductive Medicine and Gynecology, University Medical Center Utrecht, Utrecht, The Netherlands; 3 Department of Epidemiology, GROW–School for Oncology and Developmental Biology, Maastricht University, Maastricht, The Netherlands; 4 Department of Biostatistics and Research Support, Julius Center for Health Sciences and Primary Care, University Medical Center Utrecht, Utrecht, The Netherlands; Peninsula College of Medicine and Dentistry, United Kingdom

## Abstract

The onset of menopause has important implications on women’s fertility and health. We previously identified genetic variants in genes involved in initial follicle recruitment as potential modifiers of age at natural menopause. The objective of this study was to extend our previous study, by searching for pairwise interactions between tagging single nucleotide polymorphisms (tSNPs) in the 5 genes previously selected (AMH, AMHR2, BMP15, FOXL2, GDF9). We performed a cross-sectional study among 3445 women with a natural menopause participating in the Prospect-EPIC study, a population-based prospective cohort study, initiated between 1993 and 1997. Based on the model-based multifactor dimensionality reduction (MB-MDR) test with a permutation-based maxT correction for multiple testing, we found a statistically significant interaction between rs10407022 in *AMH* and rs11170547 in *AMHR2* (*p* = 0.019) associated with age at natural menopause. Rs10407022 did not have a statistically significant main effect. However, rs10407022 is an eQTL SNP that has been shown to influence mRNA expression levels in lymphoblastoid cell lines. This study provides additional insights into the genetic background of age at natural menopause and suggests a role of the AMH signaling pathway in the onset of natural menopause. However, these results remain suggestive and replication by independent studies is necessary.

## Introduction

The timing of the end of a women’s reproductive life has important health implications. An early onset of menopause is associated with a higher risk of cardiovascular diseases, osteoporosis, and overall mortality, whereas a later menopausal age may increase the risk of breast, ovarian, and endometrial cancer [1]–[4]. The underlying biological mechanisms for these associations remain poorly understood and much effort has been devoted to explain the observed variation in age at natural menopause (ANM) in an attempt to comprehend the etiology of these complex traits. The age at which menopause occurs varies between 40 to 60 years, with an average of 50–51 years in women of Northern European descent [5], [6]. Numerous studies focused on lifestyle and reproductive factors in association with ANM. Some evidence for an association with ANM has been observed for smoking, parity, and body mass index (BMI), but results have been mainly inconsistent [7]. Furthermore, the individual variation in ANM is thought to be under genetic control. The heritability estimates range from 31 to 78% [1], [5], [8]. So far, genome-wide association studies (GWAS) have identified seventeen menopause loci that function in diverse pathways including hormonal regulation, immune function and DNA repair [9]–[11]. Despite the large efforts made in unraveling the genetic background of ANM, only a small part can be explained through genetic factors identified so far. Most studies investigated the effect of only one SNP at a time, while it is obvious from biological studies that biological processes are influenced by multiple genes in complex networks [12]. Investigating gene-gene interactions might be a first step towards complex interaction analysis.

In a previous study, we investigated genetic variants in genes involved in initial follicle recruitment in association with ANM among 3445 Dutch women participating in Prospect-EPIC [13]. In that study, we observed an association between ANM and two single nucleotide polymorphisms (SNPs) in *AMHR2* (rs2002555 (β = 0.30, *p* = 0.021) and rs11170547 (β = 0.31, *p* = 0.049)), and one SNP in *BMP15* (rs6521896 (β = 0.41, *p* = 0.007)) [13]. Moreover, we found that the two SNPs in the *AMHR2* gene were associated with age at natural menopause in interaction with parity (i.e., rs2002555: β = 0.38, *p* = 0.005 for parous women; β = −0.22, *p* = 0.58 for nulliparous women and rs11170547: β = 0.41, *p* = 0.010 for parous women; β = −0.38, *p* = 0.44 for nulliparous women). In the present study, we aim to extend the previous study by exploring gene-gene interactions among genes involved in initial follicle recruitment in association with ANM. In addition, we aim to further explore gene-environment interactions between these genes and parity, smoking and BMI.

## Materials and Methods

### Ethics Statement

The study was approved by the Institutional Review Board of the University Medical Center Utrecht. All women signed informed consent.

### Study Design

Prospect-EPIC is one of the two Dutch prospective cohort studies participating in the European Prospective Investigation into Cancer and Nutrition (EPIC) which is a multi-center cohort study including 10 European countries [14]. Between 1993 and 1997, a total of 17,357 women aged 50–69 years, living in Utrecht and vicinity were recruited through the national breast cancer screening program. At recruitment, all women completed a questionnaire with detailed questions on reproductive factors, physical activity, smoking, education level, and other life-style related factors. Moreover, all women underwent physical examination and donated a 30-ml nonfasting blood sample. A full description of the study design and cohort has been published elsewhere [15].

### Study Population

We describe an extension of a previous study by Voorhuis *et al.* for which participant selection has been described in detail [13]. Briefly, women were excluded if they were pre- or perimenopausal at time of enrollment (n = 3,497), if they experienced a surgical menopause (n = 4,449), if they used hormone therapy (n = 2,161), if they were younger than 58 years at inclusion (n = 2248), when menopausal status or age was unknown (n = 1,194), or when buffy coat samples were missing or DNA extraction failed (n = 192). Eventually, a total of 3,616 postmenopausal women with a known ANM were eligible for the current study.

### Outcome Measure

ANM was extracted from the baseline questionnaire. Natural menopause was defined according to the World Health Organization as amenorrhea for at least 12 consecutive months without other obvious reasons [16].

### Laboratory Methods

Methods of blood collection, DNA extraction and genotyping have been described in detail [13]. Duplicate samples were included to assess the quality of the genotyping process. Women with a call rate smaller than 95% were excluded (n = 171). The average genotyping success rate in the remaining 3445 samples was 99.3%.

### Gene and SNP Selection

The selection procedure for genes and SNPs has been described in detail previously [13]. A total of 23 tagging SNPs were selected among 5 genes involved in initial follicle recruitment: rs10407022, rs7249235, rs733846, rs886363, rs3746158, and rs4806834 in *AMH*; rs2002555 and rs11170547 in *AMHR2*; rs3810682, rs6521896, rs17249566, rs5961233, and rs3897937 in *BMP15*; rs7641989, rs13064974, rs11924939, and rs10804661 in *FOXL2*; and rs10491279, rs254286, rs803224, rs4705974, rs30177, and rs11748063 in *GDF9*. We excluded two SNPs with a minor allele frequency smaller than 0.05 (i.e., rs4806834 and rs17249566), leaving a total of 21 SNPs. For the investigation of gene-gene interactions it should be avoided to include SNPs that are in linkage disequilibrium (LD) with each other in order to prevent spurious interactions. Therefore, we used PLINK to generate a pruned subset of SNPs considering a window of 21 SNPs, a shift of 1 SNP forward, and an r^2^ of 0.75. The r^2^ of 0.75 was advised by the developers of MB-MDR (personal communication). None of the SNPs were removed based on these parameters.

### Data Analysis

Deviation from HWE was tested in PLINK v1.07 using a *X* ^2^ test with 1 degree of freedom. We corrected for age at inclusion by using rank-transformed age-adjusted residuals for ANM (GenABEL v1.6–7). Missing genotypes were imputed using BEAGLE v3.3.2.

All possible pairwise interactions (n = 210) were investigated using model-based multifactor dimensionality reduction (MB-MDR) v2.7.5 [17], [18]. MB-MDR is an extension of the multifactor dimensionality reduction (MDR) method, a nonparametric exhaustive data mining method that considers all possible interactions between SNPs and classifies individuals into high and low risk groups [19]. MB-MDR, in contrast to MDR, is capable of analyzing quantitative traits and is able to adjust for main effects. Moreover, it introduces an additional ‘no evidence’ group. A full description of MB-MDR is available in references [17] and [18]. As suggested by the authors, we adjusted for main effects by adjusting for the lower-order effects of the SNPs in the SNP-pair under investigation using a co-dominant coding scheme. This method provides the best balance between type I error and power [17]. Multiple testing was accounted for by adopting a permutation-based maxT correction with 999 replicates.

Gene-environment interactions were evaluated using MB-MDR by including the environmental factor of interest as a categorical variable in the MB-MDR analysis. We investigated interactions between all SNPs and parity (parous (yes/no)), smoking (never, current, former), and BMI (<20, 20–25, ≥25).

## Results

Characteristics of the 3445 women in our study cohort have been described previously [13]. Briefly, the mean age at inclusion and at natural menopause were 63 (SD: 3.4) and 50 years (SD: 4.2), respectively. The majority of women delivered one or more children (84.6%). Only 34.5% used oral contraceptives. Half of women were ever smokers (50.7%).

No significant deviations from Hardy-Weinberg equilibrium were observed. Results from the single SNP analysis have been published previously [13].

Interaction results for the top 10 SNP-SNP interaction models are presented in Table 1. The interaction between rs11170547 in *AMHR2* and rs10407022 in *AMH* was statistically significant after correction for multiple testing (*p* = 0.019).

**Table 1:** Overview of the Top 10 Pairwise Interactions between tSNPs in Genes Involved in Initial Follicle Recruitment.

tSNP 1	Corresponding Gene	tSNP 2	Corresponding Gene	F-test	*P*-value *
rs11170547	*AMHR2*	rs10407022	*AMH*	18.162	0.019
rs11170547	*AMHR2*	rs733846	*AMH*	13.733	0.199
rs2002555	*AMHR2*	rs10407022	*AMH*	10.753	0.622
rs11170547	*AMHR2*	rs7249235	*AMH*	10.217	0.727
rs13064974	*FOXL2*	rs10491279	*GDF9*	9.470	0.84
rs2002555	*AMHR2*	rs733846	*AMH*	9.302	0.86
rs7249235	*AMH*	rs886363	*AMH*	8.333	0.958
rs7641989	*FOXL2*	rs2002555	*AMHR2*	7.265	0.996
rs10491279	*GDF9*	rs3746158	*AMH*	7.049	0.998
rs10491279	*GDF9*	rs11170547	*AMHR2*	7.024	0.998

Abbreviations: tSNP, tagging Single Nucleotide Polymorphism.

* P-values are reported after adjustment for multiple testing, based on a permutation-based maxT correction with 999 replicates.

We also tested the interaction between each SNP and parity (parous (yes/no)), smoking (never, current, former), and BMI (<20, 20–25, ≥25). After correction for multiple testing no significant gene-environment interaction was observed. Our MB-MDR analysis did thus not replicate the previously observed interaction between *AMHR2* and parity based on linear regression.

## Discussion

In this large cross-sectional study we investigated interactions between 21 SNPs in genes involved in initial follicle recruitment in association with ANM. We observed a statistically significant pairwise interaction between rs10407022 in *AMH* and rs11170547 in *AMHR2* after permutation-based maxT correction. No gene-environment interactions were observed between these SNPs and parity, smoking or BMI.

The present study is the first study investigating interactions between these 5 genes involved in initial follicle recruitment in relation to ANM. We previously observed statistically significant associations between the two SNPs that tag the gene encoding the AMH receptor (*AMHR2*; rs2002555 and rs11170547) and ANM [13]. No associations for tSNPs in *AMH* with ANM were found. In the present study we extended this study and searched for pairwise interactions between the SNP in the genes previously selected. We found an interaction between SNPs in the *AMH* gene and its receptor gene *AMHR2*. This might imply that complex interactions between these genes play a role in ovarian aging and thus in onset of menopause. However, this is the first report of an interaction between *AMH* and *AMHR2*, therefore, replication by independent studies is necessary to confirm these findings.

One of the SNPs involved in this interaction, rs10407022 in the *AMH* gene, is an expression quantitative trait locus (eQTL) for *AMH*, which means it influences expression levels of mRNAs [20], [21]. Moreover, this missense mutation is predicted by SIFT to have damaging protein function [22]. We have shown that this SNP by itself does not influence ANM, but that it may modify ANM in interaction with rs11170547 in *AMHR2.* Interestingly, this SNP is the only known eQTL SNP in these genes [20].

Anti-Müllerian hormone (AMH), produced solely by small, growing follicles in the ovary, is a validated biomarker of ovarian aging, as serum levels of this hormone are strongly correlated with the number of growing follicles [23], [24]. AMH levels have also been shown to be a strong predictor of time to menopause [23]. Genetic association studies might provide additional understanding of the biological processes underlying the correlation between AMH and onset of menopause. Motivated by the considerations outlined above, a more thorough investigation of the AMH signaling pathway in onset of menopause seems worthwhile. It may help us to better understand the biological processes that influence variation in ANM, a trait with many health implications.

In this study, we observed no gene-environment interactions between genes involved in initial follicle recruitment and parity, smoking and BMI. This is in contrast with our previous study, in which we found an interaction between parity and tSNPs in *AMHR2* using linear regression models [13]. This interaction was a replication of a finding by Kevenaar *et al.* [25]. The lack of replication in the present study might be attributed to either the different parameterization in linear regression (additive models) compared to MB-MDR (non-parametric) or to the very stringent correction for multiple testing used with MB-MDR. On the other hand, the previously observed interactions between parity and SNPs in *AMHR2* might be false positive findings. In fact, a clear biological mechanism for these interactions has not been found.

This large study with detailed information on exposures and outcome inevitably has some limitations. We were not able to replicate our findings in an independent population. However, by using a very strict correction for multiple testing, we tried to reduce to chance of false positive findings. Moreover, the statistical power of our study might have been too low to detect real interactions.

In conclusion, we observed a pairwise interaction between 2 SNPs in *AMH* and *AMHR2* in association with ANM. More studies are needed to provide additional evidence for a role of the AMH signaling pathway in the onset of natural menopause.
